# Supplementary material for: Signal neutrality, scalar property, and collapsing boundaries as consequences of a learned multi-timescale strategy
Source: PLoS Comput Biol. 2022 Aug 5;18(8):e1009393. doi: 10.1371/journal.pcbi.1009393 (PMC9462745; doi:10.1371/journal.pcbi.1009393)
Supplement: S1 Text — In the first, the actor-critic learning model is described. In the second, we analyse the interpretation of the strategy learned by the agent as a majority vote. In the last, we show how the model maintains high performance despite changes in the distribution of timescales. (PDF) [file pcbi.1009393.s001.pdf]

Supporting information for “Signal neutrality, scalar property, and collapsing boundaries as consequences of a learned multi-timescale strategy”

Luca Manneschi, Guido Gigante, Eleni Vasilaki, Paolo Del Giudice

1 Learning

The learning algorithm adopted is a reinforcement learning actor-critic model with eligibility traces [1]. The goal of reinforcement learning is to maximise the cumulative reward:

$$G_t = R_{t+1} + \gamma R_{t+2} + ... + \gamma^{t+N-1} R_{t+N} \tag{1}$$

where $0 \leq \gamma \leq 1$ is a discount factor that describes the tendency of the agent to invest in future rewards (here $\gamma \simeq 1 - 10^{-7}$). In our specific case, an episode ends when the agent chooses ‘left’ or ‘right’, or the maximum allowed time $T_{\max}$ is reached without a decision. Rewards are given at the end of the episode only. The reward is 1 for the case of correct decision, and 0 otherwise. The policy (the actor) is embodied by the probabilities p_a ($a =$ ‘right’, ‘left’ or ‘wait’) defined in Eq. 17, Main Text. Instead, the parametrisation defining the critic, *i.e.* the value function $V(t) = \mathbf{E}_p\{G_t|s_t\}$, is:

$$V(t) = \sum_{\tau} \left\{ W^s_\tau |x^s_\tau(t)| + W^c_\tau x^c_\tau(t) \right\} + b_v \tag{2}$$

where the weights $\mathbf{W}$ are learned alongside $\boldsymbol{\theta}$ (with $\mathbf{W}$ and $\boldsymbol{\theta}$ we refer to all the parameters for the critic and actor respectively), and we used the absolute value of the integrators because, similarly to the definition of Σ_{wait}, positive and negative fluctuations of the signal should contribute in the same way to the expected reward. For each episode, the algorithm defines two sets of eligibility traces, $\mathbf{e}^W_t$ and $\mathbf{e}^\theta_t$, for the

critic and the actor respectively:

$$\mathbf{e}_t^W = \gamma\lambda^W \mathbf{e}_{t-\Delta t}^W + \gamma^t \nabla_{\mathbf{W}} V(t)$$
$$\mathbf{e}_t^\theta = \gamma\lambda^\theta \mathbf{e}_{t-\Delta t}^\theta + \gamma^t \nabla_{\boldsymbol{\theta}} p_a(t)$$

where t is the time inside an episode, and $0 \leq \lambda^\theta \leq 1$ and $0 \leq \lambda^W \leq 1$ are the traces decay parameters (here $\lambda^\theta = \lambda^W = 1 - 10^{-5}$). The parameters are then updated according to:

$$\delta \equiv R_{t+\Delta t} + \gamma V(t + \Delta t) - V(t)$$
$$\boldsymbol{\theta} \leftarrow \boldsymbol{\theta} + \eta^\theta \, \delta \, \mathbf{e}^\theta$$
$$\mathbf{W} \leftarrow \mathbf{W} + \eta^W \, \delta \, \mathbf{e}^W.$$

2 Interpretation as majority voting

S1 Fig shows the tendency of the agent to make decisions when there is a coherent alignment of the contributions of the different integrators of the signal.

3 Distribution of τ

In this section, we quantify the dependence of the performance of the proposed agent on the number, range and distribution of τs adopted. S2 Fig shows an accuracy surface as the number of integrators (N_τ,x-axis) and the maximum timescale (τ_M, y-axis) of the distribution vary. In this case only, we did not rescale the intrinsic noise σ_I for the different models. It is evident how increasing the number of integrators always improve the performance. Moreover, accuracy is more clearly rising along the diagonal from the bottom-left to the top-right corners of the surface. Following such diagonal, we are indeed increasing the variety of time constants and the number of integrators available at the same time. This clear trend shows that the model prefers non-redundant features, defined over sufficiently diverse values of τ, when there is the possibility to 'recruit' more integrators. In contrast, to move upwards on the y-axis while maintaining a fixed number of integrators is typically characterised by a performance increase until $\tau_M \approx 10$,

and then by a subsequent decrease. The reason is that, for the usual setting considered ($\sigma_I = 0.02$ and $T_{max} = 2$ s) integrators with a timescale lower than 10 s can carry more relevant information than the slower ones. Thus, if we keep the number of features as constant, the accuracy would be higher for the region with the most appropriate integrators. At the same time, variety is always preferred when increasing N_τ.

S3 Fig reports the performance of two agents, one with the exponential distribution of τ adopted in the paper (black line as usual), and the second with a linear distribution of τ over the same range (red lines). The performance of the two models remain comparable as T_{max} (panel A) and σ_I (panel B) vary. The result suggests how the specific distribution of timescales chosen does not impact considerably the results. Thus, it is more important that the τs are sufficiently spread over different magnitudes with enough density to cover the considered range. Moreover, S3 Fig shows the performance of the agent with a single integrator (see Section 2.4, Main Text). The behaviour of the different timescales as the parameters vary (x-axis of the panels) are analogous to the ones reported in for the single integrator with optimised thresholds. However, for the case of the agent reported here, the performance are usually higher than the single timescales of , since this model can also exploit some temporal information.

References

1. Sutton RS, Barto AG. Reinforcement learning: An introduction. MIT press; 2018.
