# Supplementary figures and images for: Signal neutrality, scalar property, and collapsing boundaries as consequences of a learned multi-timescale strategy
Source: PLoS Comput Biol. 2022 Aug 5;18(8):e1009393. doi: 10.1371/journal.pcbi.1009393 (PMC9462745; doi:10.1371/journal.pcbi.1009393)

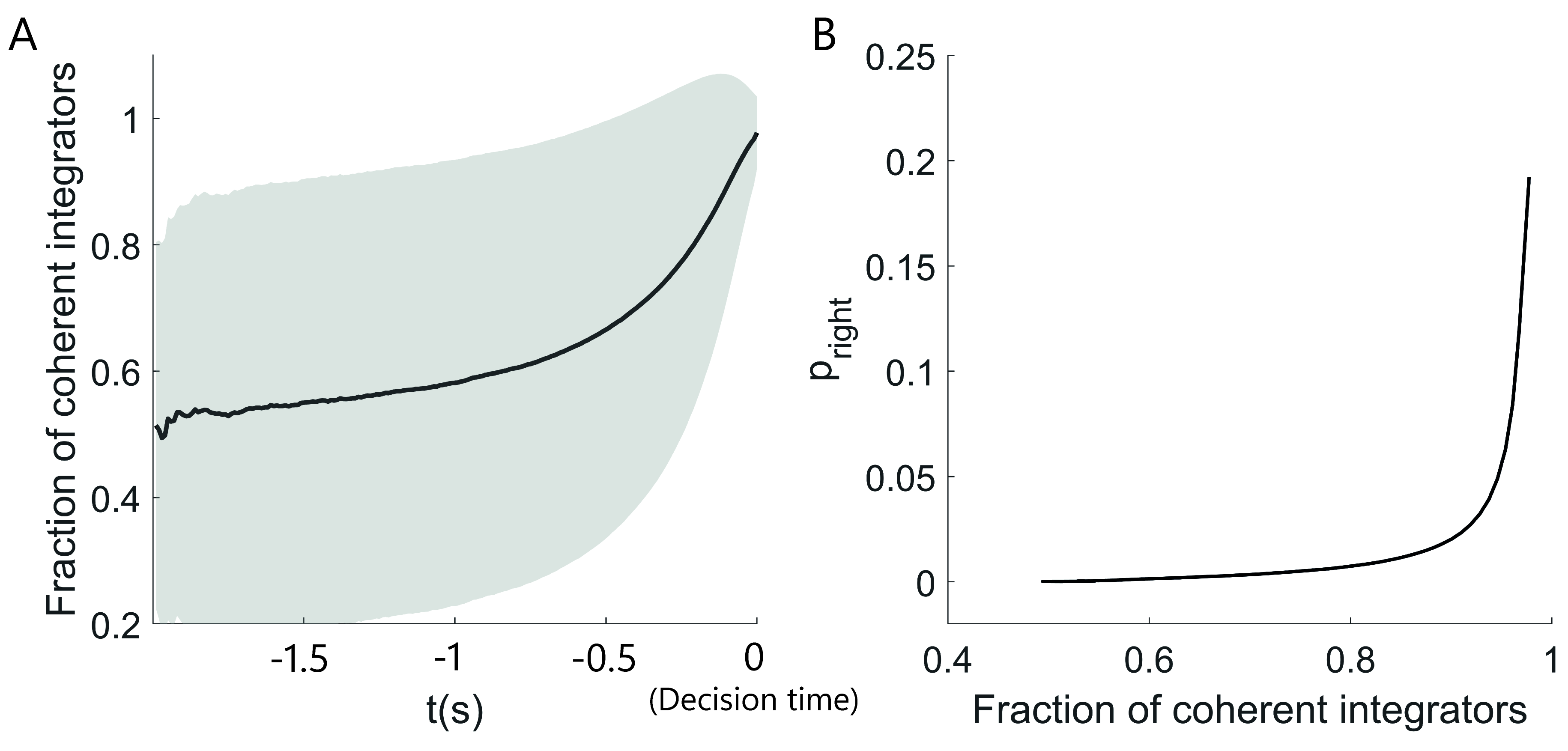

Supplement: S1 Fig — The measures are computed for the episodes where the agent correctly selects the ‘right’ action. A: Fraction of integrators that are positively contributing to the ‘right’ action. The measure is aligned with the decision time (extreme right at zero). When a decision is made, more than nine (out of ten) integrators have a positive contribution to the decision on average. B: Probability of the ‘right’ action as the fraction of positively contributing integrators changes. The probability of making a decision is considerably different than zero when the majority of the integrators align. (TIF) [file pcbi.1009393.s002.tif]

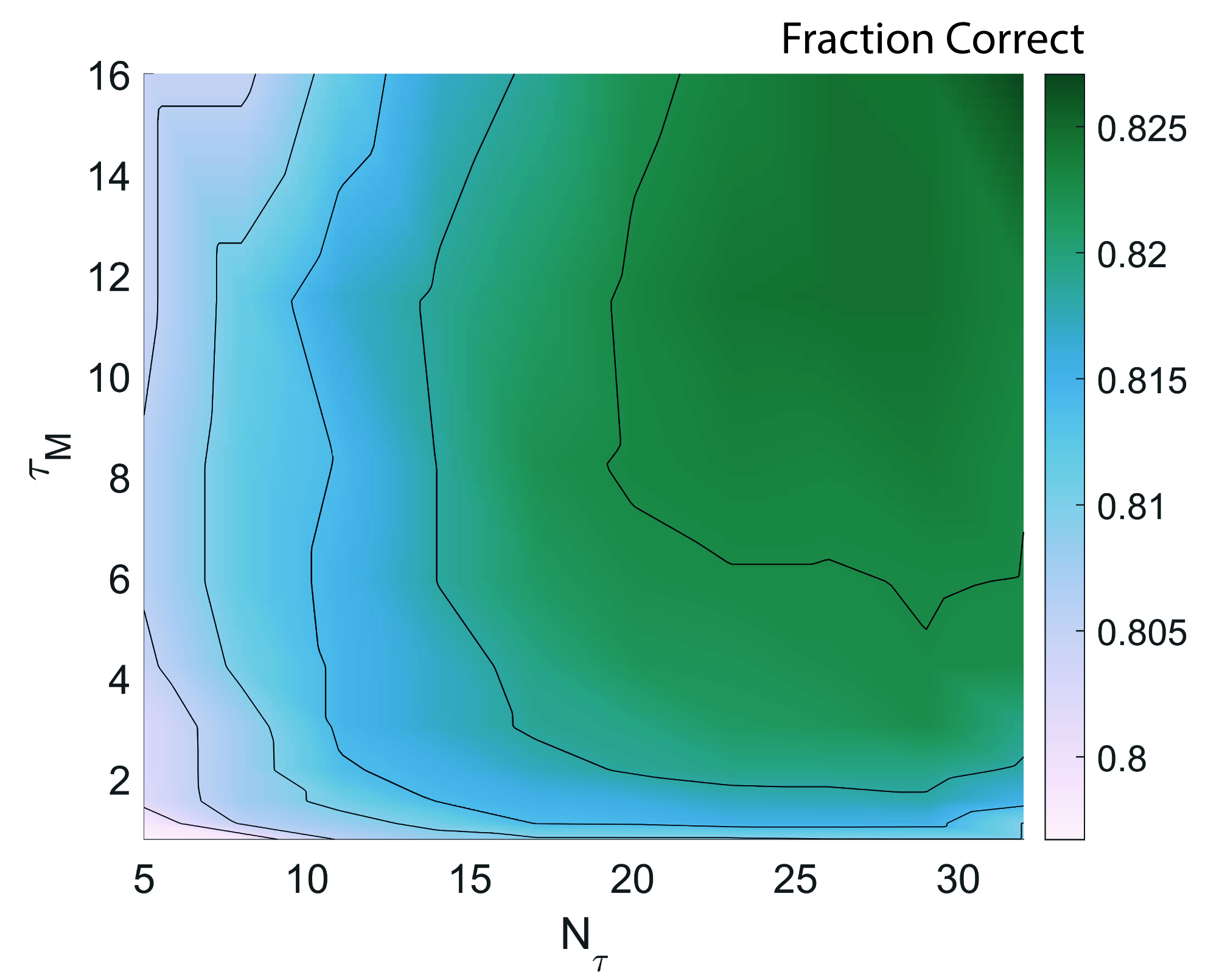

Supplement: S2 Fig — For this specific result, the intrinsic noise has not been rescaled for the different models. (TIF) [file pcbi.1009393.s003.tif]

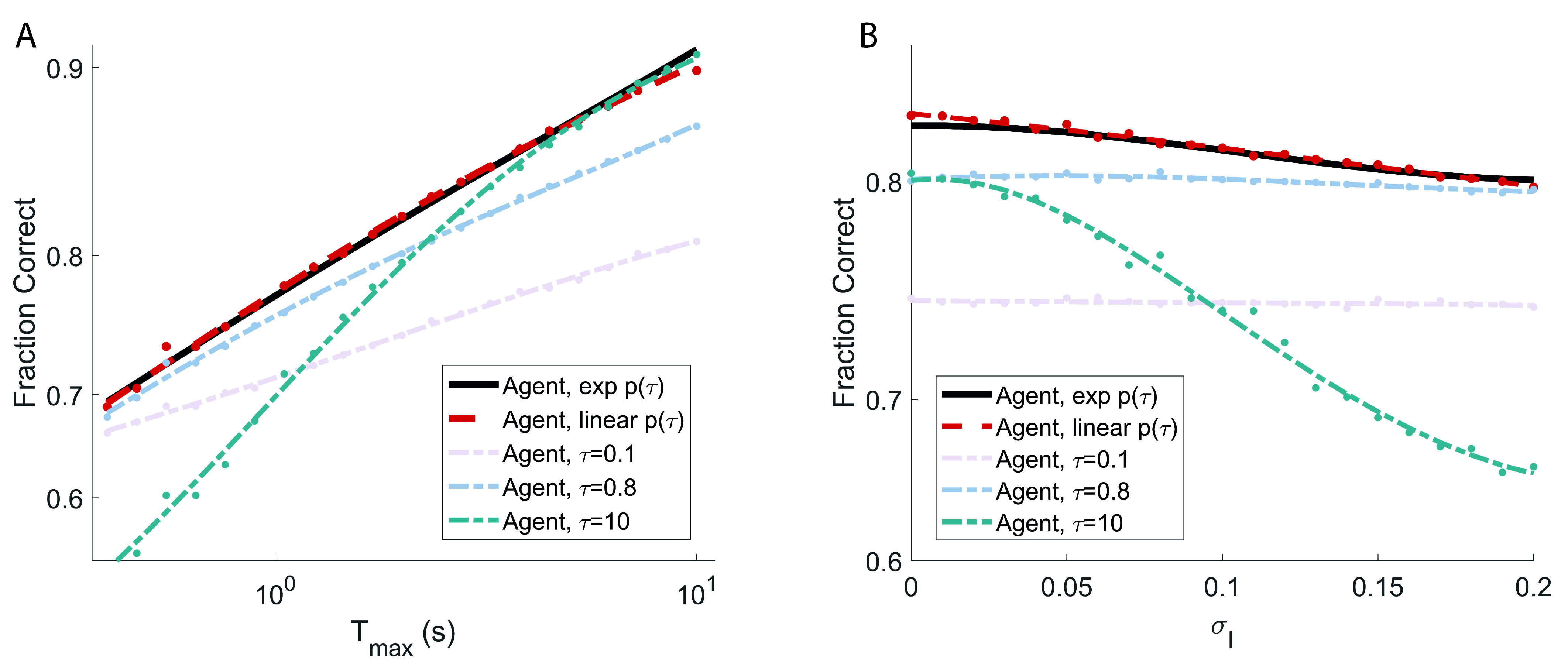

Supplement: S3 Fig — A-B: The model with a linear distribution of timescales (red, dashed line) reports comparable performance to the one proposed (black, exponential distribution). This demonstrates that the performance of the proposed agent is robust with respect to changes in the distribution of timescales, assuming that the chosen distribution has time constants over different orders of magnitudes and that is enough dense to cover the range considered. The performance of the agents with single integrators shows similar trends to the one reported in Fig 9 in the Main Text for the integrators with optimised thresholds. Thus, we refer to Fig 9 in the Main Text (Panels A and B) for more detail. (TIF) [file pcbi.1009393.s004.tif]
