# Supplementary material for: Signal neutrality, scalar property, and collapsing boundaries as consequences of a learned multi-timescale strategy
Source: PLoS Comput Biol. 2022 Aug 5;18(8):e1009393. doi: 10.1371/journal.pcbi.1009393 (PMC9462745; doi:10.1371/journal.pcbi.1009393)
Supplement: Attachment — Submitted filename: Reply_to_reviewers.pdf [file pcbi.1009393.s006.pdf]

We thank the editor and the reviewers for their helpful comments. We have essentially re-written the manuscript and clarified the contribution of each of the key ingredients of our model. Below we provide a point-to-point answer to the reviewers:

Reviewer #1

This study proposes an interesting extension of standard accumulator models (e.g. drift-diffusion models) to account in a unified framework for three behavioural signatures of decision making: signal neutrality, scalar property and collapsing boundaries. Through an agent that integrates evidence in multiple accumulators running in parallel, each one with different timescales, an alternative strategy to solve the decision problem emerges. Crucially, the authors train the agent using reinforcement learning methods to identify the importance each timescale will have in the overall integration of evidence in a simulated random dot motion experiment. The model seems successful in capturing the behaviour qualitatively (using as main benchmark previous studies, e.g. neuronal firing in LIP) and hints at some testable predictions.
The study is quite comprehensive in the description of the model and characterising its features through varied simulations. I reckon the idea in this work is novel may be of interest for a wide community working on decision making, especially those interested in computational aspects. I suggest some extra points to clarify some aspects of the model and improve the manuscript.

We thank the reviewer for their positive comments and suggestions for improvements.

1. The main alternative model to compare the proposed agent is the single-time-scale accumulator. I understand you also used the agent with multi-accumulators, but with a single time-scale, i.e. 10 accumulators with the same τ ((as in Figure 4C?). Is this correct?

We apologise for misleading the reviewer. This is not the case. We compare with a single \tau but have rescaled the noise to compensate for differences in variance. We now provide a new set of simulations demonstrating the agent, comparison with the drift diffusion model (1 timescale) and a version of the agent with (again) 1 timescale.

This alternative model was not as highlighted as the single accumulators, is there a reason for that? Your analytical presentation of the basis of signal invariance and scalar property in the model touches on the necessity of multi timescales, however showing simulations of multiple integrators with identical τ, for some of the other analyses you performed, may be helpful to support the point. This is to dispel the idea that a simple “smoothing” of the signal by averaging multiple accumulators (and not the multi-time scales) could be enough to generate the specific behavioural signatures.

We have now added a detailed methods section where we explain the model and the alternative ones. We have removed the analytical presentation because including a clock (which our new simulations showed clearly that it is necessary for obtaining the optimal decision boundary and the scalar property) complicates the analysis and we could not make a rigorous or semi-rigorous argument. Instead, we added a number of new simulations and analysed their solution. We show, hopefully beyond doubt, that the optimal decision boundary can only be achieved with multiple time scales in the clock. We also explain the solution obtained, that depends on having integrators that reach high values “fast” and integrators that reach high values “slow”, please see also reply to point 2.

2. Models as the aDDM (Krajbich, Armel, Rangel, NatNeuroscience 2010) or more recently (Jang, Sharma and Drugowitsch, eLife, 2021; or Callaway, Rangel, Griffiths, PlosCompBiol,

2021) have proposed that dynamic attentional fluctuations can affect the evidence integration process, modifying the drift rates or modulating the reliability of the evidence. Based on the premise that it is possible that parameters of the accumulation process could be modulated within a trial, can you comment on the alternative that an agent with a single accumulator but with dynamical time scales could also account for the behaviour captured by multiple-but-static timescales agent (i.e. your agent)? (static understood as an agent whose learning has finished)

We believe that these additional simulations and analyses clearly demonstrate that the optimal boundary and the scalar property are due to the multiple time scales in the clock. We refer to new figures 5 and 7. In particular for the optimal boundary we depict the reason for the initial increase and then decrease of the boundary. The fast time scales "clocks" have higher values in the beginning leading to the promotion of "wait" rather than taking the right action. Slow timescales "clocks" obtain large values later in time, pushing to a decision, see Figure 10 E, where the difference of weights for wait - right is positive for small timescales and negative for large. None of the other combinations (1 timescale with or without clock) give a boundary that is reminiscent of the optimal one.

3. One of the fundamental aspects of the model is the number of accumulators. In your case, you mentioned the agent comprises 10 integrators. It would be interesting to see in the robustness section of the results how some of the signature behaviours are affected by the number of integrators the agent has. Is there a minimum number of integrators required to generate the behaviour?

We have now added a Figure in the appendix showing how varying the number of integrators affect the solution. It turns out that the number is not that crucial for this particular problem. As long as we have timescales in the appropriate range with respect to the problem, it works. Adding more integrators only marginally improves performance, but because in this case we have not rescaled for the number of integrators as we did in the case of the single integrator, the improvement may well be due to variance reduction due to the number of integrators.

4. At the beginning of the paper you hint that this model could help with the issue of a "tuning" phase in which the agents will adjust different accumulators weights for a specific task in a context-dependent way. In this way, the same system could flexibly respond to various task demands. This is a very interesting point, especially considering that standard perceptual experiments do not deal with this stage, all those trials are usually discarded as "training" trials to familiarise the participants with the tasks. It would interesting if you can further elaborate on this point in the discussion. Since it is evident that adaptation in human observers has a completely different scale (not requiring millions of episodes as presented in the RL training), how representative could be this model to characterise that adaptation process?

We have now elaborated in the introduction (and repeat in the discussion) why we think that having multiple integrators can substitute the need for fine tuning the time constant of the model. In fact, this was one of the motivations for the work that we have now made explicit.

5. You presented details on the training of the agent to learn the weights, however, it is still not clear to me how it was implemented (e.g. you mention "a large number of episodes" but how many of them?). Was the agent trained on episodes with multiple coherence levels, with an identical number of episodes per level? Were all the different results presented for agents trained on the same episodes? In line 175 you mention that after the training, the same weights are used for the presented analysis. What were the criteria to define the end of the training??

We have now added a methods section before the results, where we quickly describe the training process and refer explicitly to the Supporting Information (and the Sutton and Barto Reinforcement Learning book) for more details.

6. About the weights, I did not find a specification of the range they are constrained to (e.g. 0

This is right, we did not constraint the weights. Now from the additional results it is clear that they can take positive or negative values.

7. The semi-analytical demonstration of the scalar property (line 342) assumes a threshold θ, however in the agent definition is emphasized that the proposed model does not rely on threshold per se. How do you connect the validity of that demonstration with the agent?

The reviewer is right pointing out the weakness of the analysis. We attempted to redo it including the clock timescales, but it was not a very successful venture. Instead, we provided additional simulations to pinpoint the role of each element in the model (please see reply to earlier points).

8. Abstract and introduction can be restructured to be clearer on the problem the model is trying to tackle. For example, in the abstract, you jump from a very general first sentence on perceptual decision evidence to a very specific second sentence on the agent with multiple timescale integrators, without an intermediate justification. In the introduction, a similar situation is found, with the definition of the features of the agent (line 32) before being clear why that agent is needed at all. This can be confusing the first time one reads the paper. Please try to revise those sections.

Indeed, we have essentially re-written the manuscript. We hope it is now clearer.

9. It may be helpful to dedicate a specific section to the semi-analytical demonstration, instead of it being part of "scalar property" section.
Please see point 7.

Extra comments:

1. *Typo in abstract "The agent discovers a strategy markedly different from the literature "standard", according to which a decision IS made when the accumulated evidence hits a predetermined threshold". (Missing 'IS').*

We have rewritten the abstract.

2. Line 41: It says "know", should it be "known"

3. Line 275: "We will focus our attention on the evolution of a key observable in the model". Is a word missing in this line?

We rephrased for better clarity.

4. Line 500: you mention: " The intermediate τ = 2.1 s, on the other hand, shows a steadier, intermediate, trend.", however in figure 9A you show τ = 0.8. Is that correct?

Yes, it was correct. However, we removed such an integrator in the comparison and added the performance of the agent without an internal clock.

5. Equation 16: I think the symbol to the right side (ς) is not defined in words (although it is understood it is the std.dev.)
6. In equation 25: I guess τ1 and τ2 represent pairs of accumulators with multiple time scales? Additionally, I'm not clear if in the right side of the equation the expression ([1 – exp(-(1/τ1 + 1/τ2)t]) continues from above or if it is equivalent to the right side of the expression above. Please clarify.

For points 5 and 6. We have removed the demonstration and added new results that show how the scalar property can be achieved by the proposed agent and not by the other comparative models.

7. Figure 4C: I think a parenthesis is missing in the description of the figure.

It has been corrected.

8. Figure 4D: this figure is a bit confusing since the numbers increase from top to bottom for the Fraction Correct. Is there a particular reason for that? Also, the lines indicating the agent performance get lost easily with the margins of the figure. It may be a good idea to separate these figures for better visualization.

We have now split Fig. 4 into two figures (Fig. 4 and 5) and adopted a standard notation for the axes.

9. Figure 8: given that you mention that the dot marks the decision time, it is hard to tell if you refer to the big highlighted dot or all the dots that seem to describe the trajectory of the accumulator. Please clarify.

We are referring to the bigger dots. We have now clarified this in the text.

10. Figure 9D: please make bigger the coloured circles in the legends, it is hard to tell which is which.

The dots are now bigger.

11. In figure 10, the colour of the legend is very light in comparison to the lines in the figure. It may be a good idea to use the darker version of the colour to ease the identification.

We have changed the legend accordingly.

We thank the reviewer for all the suggestions.

Reviewer #2

Overall evaluation

The authors attempt to consider an algorithmic (semi-mechanistic) reinforcement learning (RL) model to explain behavior and neural activity observed in a wide class of experiments

that require estimating the underlying state of a noisy sensory signal. These experiments have been widely modeled using descriptive mechanistic approaches—specifically the class of accumulator models. The primary contributions here are developing an algorithm that is implicitly close to those used to solve partially observable Markov decision problems, though the explicit relationships are not sufficiently discussed. Further, the authors draw on evidence from behavior and neural data that multiple time scales are used in the brain and that using a reservoir of integrators with multiple time constants they can capture a number of behavioral and neural features observed in experiments. The contributions include a novel RL model, connecting multiple strands in the literature, and connecting the standard integrator models to the normative RL framework. However, the current manuscript does not lay out clearly many of the connections and doesn't clearly demonstrate the robustness of their approach to the larger class of problems—one of the claims they would like to argue for. That said, I think the core proposal is a significant contribution and with further clarity and some additional characterization would be a valuable addition to the field.

We thank the reviewer. We felt it is difficult to both explain the model well and apply it to multiple tasks, so given the reviewer's statement that the contribution is appropriate, we went for the former. We expanded the model description and the analysis. We believe that the manuscript now provides a clearer description of the work and explanation of the results.

Major Comments

One of the major problems with the paper is that it tries to connect a number of important areas of research without clearly establishing the context and connections. While this doesn't impact the core proposal embedded in their model, it significantly reduces the impact and clarity of the work. For example, the authors never define or clearly connect to the formal setting for studying uncertainty in RL (and related fields) when the true state of the world is hidden and you only receive noisy observations—the partially observable Markov decision problem. While they mention (and cite) the idea, they are proposing a RL implementation without connecting to this important literature. Another example is that their model is isomorphic to multi-agent RL models, often used to study decision making in economics and computer science. (In this case, each agent would have a different time constant and the arbitration would be via the softmax.) On the biology, they don't clearly describe the literature on the variation of time constants observed across the brain (e.g. Murray et al., 2014; Bernacchia et al., 2011; Rossi-Pool et al. 2021; etc.) or inference over time for the 'clock' processes (so many it's hard to choose, but in the RL context, Mello et al 2015). If the authors appropriately frame and contextualize their work, it would reduce the sense that this is a somewhat arbitrary model that captures a few behavioral and neural phenomena. Similarly, the relationship between the normative and descriptive could be clarified. There is significant work relating RL models of decision making to the drift-diffusion class of models, including exploring a normative source for collapsing bounds (e.g. Mendonca et al., 2020; Tajima et al., 2016; Stine et al., 2020). Overall, despite the extensive literature cited, the authors need to significantly improve their background description of the problem and the relationship between the biological data, descriptive and normative models.

We thank the reviewer for their comment and the suggested literature. We have essentially rewritten the introduction and better motivated the modelling. We also included citations to Bernacchia et al. 2011, Mello et al. 2015, Mendonca et al. 2020, and Drugowitsch et al. 2019 (we already cited Stine et al. 2020, although in a slightly different context).

To highlight the link to POMDP, we have included the following in the introduction: "Our setup has similarities to a Partially Observable Markov Decision Process with opportunity costs. The agent cannot access its state consisting of the signal sign and the clock. Instead, it has access to several observations at each timestep. These observations are continuous

variables that integrate noisy information about the state in terms of signal information and the time passed. These observations progressively correlate with the agent's ``true'' state as the integration filters out the noise. The option to defer this decision in case of insufficient evidence complements the desirable action to find the sign of the stimulus. Yet, the presence of a time limit effectively imposes a cost on deferring the decision to accumulate more evidence."

A second major concern is that the authors don't provide a clear insight into the decisions that inform the structure of the model. Despite the general insight that using the reservoir of time constants in the brain could provide a general and robust mechanism for solving POMDP problems with opportunity costs (e.g. limited integration time, or fixed length sessions with variable trial lengths, etc.), the model itself is presented without specific motivations for the structure or relation to prior or alternative models. While I don't feel that the authors need to formally compare their model to alternatives (unless they found it valuable to place it in context), the choices they make feel too arbitrary to the reader and they don't show how the performance varies under different parameter regimes or reduced versions. This doesn't provide a sense of the generality or generalizability of their proposed model in the larger context. Specifically, there are a few key questions that should be addressed. Most of these are relatively straightforward to answer or provide the necessary details.

Besides adding a clear description to motivate the choices, we have added new simulations that make clear to which model elements the properties we observe depend. We hope this provides a clear justification for the elements and their interpretation. We believe the model can be applied to more general tasks (for instance possibly machine learning problems with time-varying inputs or replacing the integrators with a reservoir), but decided it is beyond the scope of this paper.

1) Why is it necessary to include a separate time signal? While it is clear the model uses the clock-like integrators to facilitate the speed-accuracy tradeoff, it's unclear how the model would perform without it in different contexts. Also, it's unclear why the model cannot extract this time signal from (a transform or function of) the primary integrators which have similar dynamics. Generalized clock-like information is actually relatively unclearly available in biological systems outside of very specific functions (e.g. circadian clock). The kind of temporal inference used in Mello et al., 2015, for example, proposes that general dynamics of sensory and behavioral processing can be used to infer timing and shows the scalar properties required.

We have now explained how the clock shapes the decision making boundary, and depicted how this particular solution emerges, please see response to Reviewer 1, Point 2. Our simulations suggest that the scalar property may be a consequence of the decision boundary, which in turn critically depends on the multiple time scales in the "clock".

2) The relationship between the formal state model, training regime and results. Specifically, often the speed-accuracy trade off is affected by the opportunity to make a rapid choice—even if it's likely to be incorrect—if the subject can move to a new trial where the difficulty drawn might be easier. Often, this is often controlled for in the primate work cited, but the generality of the algorithm should reflect this as the RL framework is interested in future discounted or average reward rates. Also related to this is a clearer description of the state-transition model used and assumptions about its relationship to time. Classically, RL models have often used tapped-delay lines where time is explicitly in the state space. Here, using eligibility traces, it's unclear if time is also in the states or not.

Regarding the speed-accuracy trade-off, this can be modeled by changing the maximum allowed time to make a decision or the gamma factor in the RL algorithm. Indeed, for a lower maximum allowed time the model needs to react more rapidly. Waiting more time means accumulating more information, but also increases the probability not to make a decision in time. This trade-off can be consequently found when we vary T_{max} (Fig 9 A-C).
Another consequence of this can also be seen in the new Fig. 10, where at the beginning of training the agent reacts quickly during an exploration phase. In such a regime, the agent understands that guessing randomly is better than waiting too much time. However, in our simulations, the difficulty of an episode is always sampled randomly at the beginning of the latter.

Regarding the definition of the features, our agent at time t has access to the activities of the integrators of the external signal and of the integrators of a constant over multiple timescales at that time. Thus, time is encoded in the latter integrators, which measure its passage over multiple timescales. In particular, an integrator with an extremely high value of tau (in comparison to the time scale of the decision; in our case this would be tau=10 s) can be considered as a counter of time. In other words, the integrator with tau=10 s is analogous to giving the explicit temporal information to the model. However, we note that all our features are noisy.
We analyse now in more detail the importance of this perception of time in Fig. 8, which shows how our model discovers the optimal decision boundary (or rather a close approximation of it) thanks to the **multiple** timescales that encode temporal information.

3) A clear prediction that often fails for standard drift-diffusion models is the distribution of error times. The authors show a reasonable (compared to data) error trial reaction times, but don't differentiate this from drift-diffusion models that don't account for them.

This is a great suggestion. We have now explicitly compared and demonstrated that the scalar property cannot be reproduced with the drift-diffusion model or with our agent with a single timescale.

4) How are the parameters chosen and how robust are they? For all the parameters not derived from data or where the values are explained, how did they get fixed and how do they affect the model's performance. For example, the discount factor, eligibility trace decay, learning rate, etc. Further, if the time constants were chosen to be linear rather than exponentially distributed, how would the model perform? How many time constants are needed in the reservoir? How are the time scales of the perceptual information rate and the reservoir related?

We have elaborated on the description of the model. We also followed the suggestion of changing the distribution of the time constants with no changes in the results. We have now included a Figure in the appendix showing how varying the number of integrators affect the solution (see also point 3 for Reviewer #1).

5) The explanation for the consensus periods which lead to the signal neutrality are not sufficiently explained or explored. As the authors point out, other mechanisms can lead to convergence at the time of choice in neural networks and dynamic models. However, the authors want to claim a relationship between the integrators, effective collapsing bound (decision time) and consensus in the weighted integrators. While intriguing, it's unclear both how reasonable the mapping is between their model's integrators and the LIP function and activity. Further, would the distribution of errors in time given the 'windows' of consensus predict distributions of errors (or choices) uniformly across the trial? If the weights produce different effective balance of short and long time constants at different points in the trial, it seems the distribution of choice and error times might be non-monotonic and different, at

least from the description and example plots provided. Finally, more general statistics on the consensus periods across runs would be helpful.

We have now elaborated the link of the model ingredients to each of the properties. In addition, we have added a new figure to demonstrate statistically how the agent makes decisions when there is an alignment in the contribution of its integrators (Fig. 11, Supplementary Information).

6) A clearer (i.e. likely a figure) to understand the weights distribution through learning and in relation to the integrators at decision time after training would be helpful. The claims about the dynamics require an understanding of the learned weights that are not clearly presented (or how they might vary in the different experiments run).

This is a great suggestion which led to the interpretation of the role of the clock in shaping the decision-making boundary. We have added the figure and appropriate comments in section 3.4. Practically, we show how only the agent with explicit internal clock information can reproduce the collapsing boundaries.

7) The code available is uncommented, terse and without generation of the plots, and not provided in a reproducible format (e.g. without specific versioning of libraries, etc.). This should be fixed and, ideally, provided in a Jupyter Notebook where readers can manipulate the model easily. Ideally export the corrected GitHub repository and code to a Binder so that it is fully reproducible.

We now provide on GitHub also a Jupyter Notebook where the most important results are reproduced: definition and training of an agent; retrieval of the parameters of an agent at a given time during the training (for example, to find the evolution of performance); a simulation of an agent with a distribution of mus and estimation of performance and response times statistics; a plot of the weights; computing the “Shadlen’s measure” (and making an illustrative plot akin to Fig. 4A); computing and plotting the collapsing boundary.

Two additional (but less critical) comparisons would be of significant interest, despite not being strictly necessary to solidify the current work.

1) Extend the model to capture the work on free choice with opt-out (e.g. confidence based) decisions, where the subject has the option to select a lower—but certain—payoff. The POMDP framing and connecting to this line of work on the relationship between these perceptual decisions, confidence and DDMs (e.g. Drugawitsch et al., 2019, etc.).

We apologise to the reviewer that we haven’t addressed this point. We felt that with the additional analysis now provided, the paper has become perhaps too long, and an extension would decrease the accessibility of the manuscript. Instead, we comment about this specific suggestion in the discussion.

2) The model’s relationship to analogs of the ‘identification’ and ‘categorization’ task from Mendonca et al., 2020 where the source of noise and effects on reaction time vary depending on the effects of learning related noise vs external sensory limited noise.

We added this to the discussion, together with the point above.

Minor comments

The authors would benefit from a simpler and less difficult to read notation. The use of the same variables with similar sub or superscripts vs clearly separable variables makes it difficult to quickly and clearly read the details without lots of checking and extra effort. There are also errors (I believe) in references in places to various variables (e.g. when referring to the different weights in equation 35 and the text in the following sentence seems to be missing a superscript and tilde accent). In general, to facilitate readability by a broader (cognitive or neuroscience) audience, simply making the notation clearer and grouping the details in the methods except where core to the points would be helpful.

What is the formal assumption about the form of the memory (the integrators) in the model with respect to the standard assumptions of RL? Normally, RL solutions are limited to (PO)MDP assumptions where the agent does not explicitly store information in a form that breaks the Markov assumptions (e.g. model-based and model-free approaches commonly used don't incorporate information from prior states in their policy or value function calculations—except during learning). Here, it's unclear how exactly the authors are thinking about their integrator processes with respect to the standard (PO)MDP framework.

We are thinking of the integration process as the equivalent of state augmentation done in some problems that by definition may not be Markovian, but once history is viewed as a state, they become Markovian. Every observation contains the whole signal history, therefore it shouldn't matter how the agent arrived at it. Another way we can think about it is that the observation is correlated to the state and time P(o|s,t) but the time effectively is denoising the observation, improving its quality rather than breaking the markovian property.

The relationship between the agent training vs subjects seems unclear. In general, human performance on this class of perceptual decision tasks asymptotes in a short period of time (a few days) where non-human primates require months for similar performance. Presumably, these differences are not related to the actual number of episodes, but rather the ability for humans to understand the structure of the task and goals explicitly, while the primates learn this (via trial-and-error) along with task improvement. A more complete characterization of the learning of the agent and explanation of the behavior in early learning vs asymptotic vs humans and animals might help. Or, simply characterize the agents learning and generalization but don't relate it to the experimental data—as the mapping is likely complex.

The proposed agent is intended as a model for animal learning (purely “model-free” trial-and-error learning) and thus it neglects all the complex features of what often happens in human subjects. In this respect, we underline how the number of training examples the agent needs to reach a nearly-maximal performance is not, in very general terms, too dissimilar to what has been found in typical animal training. Indeed, our agent also starts the training being completely unaware of the nature of the task.

Although we show what happens in the agent during learning, we do not claim that the trajectories of, for example, performance and response times, as the training proceeds, can be quantitatively or even qualitatively compared to what happens in animal subjects: we consider this point outside the scope of our model, at least at present.

Clarify the framing for optimality (e.g. in the text in lines 468-72) where optimal performance is determined by the objective or cost function assumed. In many of the models, these don't include aspects like generalization across task variations, etc. The authors seem to want to claim an approximation of some optimal behavior, but it's not clear if the model is actually

specified to be optimal for a more general class of problems or what the assumptions are in the related models and literature they cite and discuss.

In the cited passage we qualitatively compare the behaviour of our agent (more specifically, the non-monotonic behaviour of the decision boundary) to what Drugowitsch et al. 2012 found to be the optimal, as a solution to a very similar task. Thus, there we do not claim that our agent is optimal, but rather that, through reward maximisation and the presence of multiple-timescales, our agent is able to find by trial-and-error a strategy that seems to be congruent to the theoretically optimal one.
More in general, when we say “optimal” we refer to the most basic meaning of the word in this context: the strategy that gives you the highest performance (and, equivalently for the agent, the highest reward). Yet, in a broader perspective, we argue that our agent - limited to the present task (with of course the idea that this could possible generalise to other similar tasks as well) - not only finds a very good strategy, but that that strategy is robust to variations in some of the basic parameters defining the task itself (it is “robust”). And that this property (“robustness”) is a desirable property of a biological plausible learning agent.

What is the relationship to the proposal of leaky (and possibly adjustable) integrators (e.g. Ossmy et al., 2013)?

We thank the reviewer for the interesting paper, which is now cited. The key difference lies in the fact that in our model we have multiple timescales available at any time, both in the integration of the signal and in the perception of time. We note how the majority of the interesting features that we observe are a consequence of this availability, and are not simply reproducible by selecting a single, despite optimal, timescale.

Reviewer #3

Prologue
I thank the editor and authors for the opportunity to review this interesting paper. Although I enjoyed reading about the idea, the process was made difficult with the figures all at the back of a very, very long manuscript. For future revisions please insert the figures where they belong along with the legends within the main text.

We thank the reviewer for their positive comments. We concur that the manuscript was difficult to read, and we have now re-written significant parts of it, added new results and changed its structure. We hope that the model and our claims are now clearer.

Summary

The authors developed a perceptual decision-making model which consists of multiple time-scale accumulators as compared to the typical single time-scale accumulator model such as the drift-diffusion model. The authors trained this model through reinforcement learning, so that it can come up with an effective decision-making strategy which can deal with highly volatile environments. Using simulation, they find that the model produces typical features similar to experimental findings from single-neuron decision-making studies such as signal neutrality, scalar properties and collapsing bounds. The authors attribute the manifestation of these properties to the multiple time-scale accumulator structure, suggesting it to be a critical feature for decision-making.

The topic is relevant and interesting, and the idea of a multiple, parallel time-scale accumulator structure in the brain working together to deal with the temporally dynamic environment sounds intuitively plausible and intriguing. The concept of multiple time-scales

in human perception is also important and has gained much attention, therefore expanding a typical accumulator model to incorporate multiple time-scales seems timely. The model seems original and interesting as it attempts to focus on key features observed in real data. However, I find the paper not so easy to comprehend and thus it can be improved in terms of writing and presentation, as well as elaborating more on why their model and its replication of some features in experimental data is so critical in understanding perceptual decision-making. Additionally, some of the results seem to warrant more explanation. Crucially, without testing the model on real data, I find the paper to be somewhat incomplete, albeit with potential. Or at least if the simulations were done for different kinds of simulated tasks dealing with stimuli with actual multiple time-scales, perhaps it can highlight more the strength of the model. Below are specific comments and questions about the paper.

We hope that the major re-writing and additional experiments have significantly improved the paper. We have not extended the model to different problems as this would make it a particularly lengthy paper, given that it is now augmented by its initial stage. Instead, we hope that the demystification of each part of the model will convince us that in principle the model can be applied to other models. We also now consider using this model in machine-learning scenarios, but this would require a different type of manuscript.

Introduction-related comments

In page 5, the authors write that signal neutrality, scalar property, and collapsing bounds are the hallmarks of an optimal strategy in decision-making. However, they do not explain why this is. It seems the authors are basing the validity of their model on showing that these three properties can be reproduced from the proposed model, specifically due to the multi-time-scale accumulators. Therefore, it seems critical to first convince the readers that these properties are indeed crucial to optimal decision-making in the introduction (i.e., L118-L121: sentence difficult to understand).
This comment matches comments from previous reviewers #1 and #2. With the newly written introduction and additional results we hope that all these are addressed.

Results/Methods-related comments

2.1 Model description: It seems the random dots' coherence is modeled as the mean value of a signal with mu. It says the value of mu is resampled for each episode. Was the distribution continuous? How many values could mu assume throughout the training? If the mean was zero in a gaussian, then most of the trials were quite difficult? How did this affect the training?

If the model's strength is to have multiple time-scale accumulators, why wasn't the input stimuli composed of multiple time-scales as well? For example, using different sampling rates of the signal or using signal of which coherence is changing within a trial, etc. may be able to highlight the model's strength more.

We now explain the importance of the timescales in signal integration in terms of robustness and with the added benefit of not having to return a parameter. In fact we do have different signal coherences in the episodes, we only separate the coherences for the analysis. We

apologise that this was not clear in the first draft, and we hope it is now clear. The role of the timescales in the clock is, instead, critical for implementing an optimal-like decision making boundary, see Section 3.4 and its corresponding results.

The symbols and variables are not explained at first appearance; xi should be explained at L152, not L160. What is the role (meaning) of b?

Xi has now been introduced before and explained accordingly.
The terms b are biases, which can be described as the tendency of the agent to make the corresponding action 'a priori'. For instance, b_{right} reflects the propensity of the agent to make the 'right' action before the start of the episode. We have now made this clearer in the text.

L165: In the figure, the white circles alone do not seem to be enough to explain the softmax combination. A more descriptive cartoon may help. Same for V(t) in L185-L187. The figure doesn't add much information about what the text is meaning.

We have now reworked the figure and applied some changes to emphasize the summation across different integrators and the softmax policy.

2.2 Decision as a majority vote: this part seems to be key to highlight the novelty of the model. Yet, how the leaky integrators with such different time-scales align at the same time point to create bursts of probabilities is not clear. How does a 10 s accumulator already have such high probability after only 400 ms after trial onset? (point 2 in Fig 2). Also, from Fig 2 it seems decisions are made at points 1 and 2. Does this mean there were two decisions within an episode of 1.6 s?

To analyse the decision as a majority vote was an insight to interpret the model's behaviour. We can now statistically demonstrate how the agent makes decisions when there is an alignment in the contributions of the integrators (see Fig. 11). In Fig. 2, the reviewer is correct, and an integrator with 10 s cannot have high activity at 400 ms. However, in the figure we show the integrators' strength (coloured circles) related to their baseline activity, and not related to their absolute values. For instance, when a circle is red, it means that the corresponding integrator has a higher value in comparison to its mean value at that time. Thus, the integrator with 10 s did not peak, but had a value of activity greater than usual.
In all episodes, there can be one decision 'right' or 'left' only. At point 2, a decision has not occurred, but the probability to make a `left` decision was greater than zero.

Figure 3C: What are the white dots? In the text it says that the parameters of the curve were fitted to experimental data. Does this mean the authors obtained experimental data from reference 4? It is not clear what actually has been done to produce Figure 3C. Please clarify.

The dots correspond to the model, while the black trend shown is taken from the experimental paper, where they reported the result of their fit. We have used the result of their fit, i.e. the parameters, to reproduce the function reported in black.

Figure 4D: Figure shows that agent shows the exact same signal neutrality and accuracy for all taus. What does this mean? Please explain.

The agent is only one and exploits multiple integrators. Thus, we have reported its performance with a horizontal line. In contrast, the other results report the performance of a single integrator with optimised threshold or of an agent with a single integrator varying tau. For clarity, all the models used are now defined together in a Methods section at the beginning of the paper.

In section 2.5, the authors show that the scalar property and signal neutrality both share the same origin, namely the multiple time-scales present in the model. It would help the reader not so familiar with the technical derivation if there was a simpler explanation why multiple time-scales leads to both these features (L387-L390).

Unfortunately, we don't have a simple explanation for the reason why signal neutrality and the scalar property arise (we have also cut the "loose" theoretical argument we provided, since it was not well founded - see also point 7 of reviewer 1). However, we have now added simulations to demonstrate more precisely what the ingredients to reach such results are. For instance, the new Fig.6-8 show how multiple timescales on the internal clock mechanism are necessary.

Figure 10A: What is the reason for the four time-courses suddenly diverging before 10^2?

Before the diverging point, the agent is not exploiting the different integrators. Indeed, a strategy where the agent responds in the same way regardless of the task difficulty can not depend on the integrators of the signal. By definition, the activities of the later integrators would vary for different task difficulties. Thus, the strategy of the agent would be 'coherence-dependent' if it was exploiting such integrators.
After the diverging point, the agent starts to understand how to accumulate the information coming from the external signal and the different trends differentiate.

Writing-related comments

I think the writing can be overall improved to be clearer and simpler, by using plainer sentences and easier words, considering many if not the majority of readers are probably non-native speakers.

Here are some examples with suggestions in red font:

Second line of the abstract: "We endow..", this sentence can be simpler, e.g., "We introduce/developed a probabilistic agent comprising .. with a wide range of time-scales.. to weight differently filtered versions of a noisy signal". Then the next sentence, ".. according to which a decision is made when the accumulated evidence hits..". Also the next sentence "The agent instead decides during short, transient windows, when these multiple integrators line up, similar to a majority vote". Maybe this sentence and the previous sentence can be combined, to make it clear what the author's new model does differently than the standard accumulator model. Also, the next point 1): here, the authors write 'signal coherence' without first saying anything about the task, therefore what coherence means here is vague. Point 2): perhaps an easier word such as clearly instead of 'glaringly' would be better. Again, authors talk about signal coherence without introducing it. Simply including a short sentence about what task has been used before introducing what the new model can do would help. Second to last sentence of abstract: ".., which emerge from the interaction of a multi-time-scale learning agent with a highly volatile environment, are hallmarks of an optimal decision strategy ..". There are many examples like this throughout the text, and I recommend trying

to make the sentences plainer and clearer for the reader, since the content itself is already quite technical.

Also, some sentences (e.g., L556-L558) seem to be incomplete, without a subject. These need to be re-written in order to accurately convey what the authors would like to say. In addition, often the authors cite a paper without sufficiently describing the citing content (e.g., L584). It would help the reader if the text is written more independently of the references.

We thank the reviewer for the careful reading of the manuscript and the suggested improvements. We have extensively rewritten the text, notably abstract, Introduction, and Discussion.

Editing-related comments

- *Please choose either timescales or time scales (or time-scales).*

We have chosen timescales for all the text now.

- *I think putting an 's' after the x(t) to indicate plural is strange, since x(t) is not a word. Perhaps simply x(t) may suffice.*

We made this (admittedly not utterly elegant) choice just in order to clearly convey to the reader that the variable x represents an array of values and not just a single variable.

- *Figure 1: it seems the two x(t) in the figure are flipped).*
- *L430: then -> than. More cases like this throughout text.*
- *Figure 3: 'agent' and 'model' seems to be used interchangeably. If that is the case, please choose one, since it is confusing.*

We are aware that this choice can be somewhat confusing; yet we weighed this risk against having too many repetitions that would make the text more difficult to read.
