# Supplementary material for: Signal neutrality, scalar property, and collapsing boundaries as consequences of a learned multi-timescale strategy
Source: PLoS Comput Biol. 2022 Aug 5;18(8):e1009393. doi: 10.1371/journal.pcbi.1009393 (PMC9462745; doi:10.1371/journal.pcbi.1009393)
Supplement: Attachment — Submitted filename: Response.pdf [file pcbi.1009393.s007.pdf]

Reviewer #1

The authors have made good work addressing the points and improving the manuscript. I would also like to highlight the quality of the figures. I am making some final extra suggestions just to clarify some points in the latest version:

1. In line 42 you state that "To avoid the issue of tunning a single parameter to the data…", however, I think that at that point of the introduction it is not fully clear yet why it is an issue at all (especially for someone outside the field of decision models). It may be a good idea to restructure that paragraph, so the disadvantage of a single parameter is clear. Maybe moving the paragraph on the biological evidence supporting multiple timescales ahead can help.

We rephrased for better clarity, emphasising the link with the previous discussion.

2. Thank you for including extra information on robustness and the number of integrators (section 5.2). It would be great if you can add a plot similar to Figure 12, not only for fraction correct, but that also shows how signal neutrality and scalar property are affected by those two factors.

We plan to study this in greater detail in the future. Unfortunately, producing a statistically relevant result for signal neutrality and scalar property for each point in the plane is computationally very expensive (take for example Fig. 5 and Fig. 7: to get those results - basically one point in the plane, we are averaging 100 repetitions of the entire training - this alone took several hours of computation!).

We thank the reviewer.

Extra comments:

1. In the abstract, in the first line, I'm not sure in which sense the word "underline" is used.

2. In line 8, when it says, "on the other hand", it is not clear what you are contrasting.

3. In line 37: "The task is reminiscent of the famous Shadlen experiments…" please add the reference.

4. In subtitle 2.2. typo "Relationshipt"

5. In line 292: "The comparison with the fixed-t observer sheds lights", should be "sheds light"

6. In line 309, it is mentioned that in Figure 3b, the agent is represented by a dashed line, when there is no dashed line in that panel.

7. In line 364, again it is referred to a dashed line in figure 5, when the figure does not show it in that way.

8. Line 484: typo "such a non-monotinic trend…"

9. Line 484: typo "demonsrated "

10. Line 508: typo "asimptotically"

11. Line 639: typo "scaler property"

12. In the supplemental information: Typo in the first line, page 2: "In cotrast"

13. In the paragraph of the second page of the Supplemental info: "Fig. 12 report the performance of two agents, one with the exponential distribution of τ adopted in the paper (black line as usual)…". I think the description does not match the figure. Should it be Fig 13 instead?

14. In Figure 2, the caption does not include a description for s(t).

15. In Figure 2, typo in "spceific "

16. In figure 9D: Thanks for making the circles in the legend bigger. However, it may still be difficult to identify the colour of the circles in the plot. Maybe, increasing slightly their size or removing the margins of the dots can help.

17. In figure 10: from just looking at the figure, it may take a while to notice that the grey lines correspond to specific episode numbers. It may help to add the episode number as a legend for the panels D-E-F panels or use more distinct colours for the lines.

We really thank the reviewer for the careful reading of the manuscript!

We addressed all the points.

Reviewer #2

Overview

The authors consider an algorithmic (semi-mechanistic) reinforcement learning (RL) model to explain behavior and neural activity observed in a wide class of experiments that require estimating the underlying state of a noisy sensory signal. These experiments have been widely modeled using descriptive mechanistic approaches—specifically the class of accumulator models. The primary contributions here are developing an algorithm that is implicitly close to those used to solve partially observable Markov decision problems and that suggest a reservoir of time constants better explain features of the experimental data than other (sometimes more normative) approaches proposed. The contributions include a novel RL model, connecting multiple strands in the literature, and connecting the standard integrator models to the normative RL framework. The revised manuscript addresses the majority of concerns highlighted by the reviewers in the first round. The manuscript still has a few areas where some simple improvements would likely increase the readability and impact, and the code provided should still be commented more substantially. However, these problems should not prevent publication.

Comments

The manuscript is vastly improved and most of my major objections were well addressed and I believe those of the other reviewers. In many places, the text is more complete and the

additional model comparisons address a number of the questions open in the original manuscript. I also think the authors did a good job in responding to the reviewers' questions and suggested changes and the additional supplementary information is valuable. I believe it is acceptable for publication, but I have a number of comments that could improve the manuscript and clarify a few remaining questions.

One example is that the core operational definitions for the criteria used to evaluate the model and its relationship to the prior literature could be introduced more clearly. The term "signal neutrality" is used both as though it is a common technical term in the manuscript and in a way that is confusing to the reader. The other two criteria are defined in terms that are common in the field and well understood casually, though the use of 'scalar property' without mentioning Weber (scaling) and the large related timing literature directly in a manuscript targeted to a wider audience is a major oversight.

We now underline, already in the Introduction, that "signal neutrality" is a shorthand we use to point to a specific experimental finding. We have reformulated the "experimental" definition of signal neutrality (always in the Introduction), and added two relevant references.

We thank the reviewer for mentioning Weber scaling, a term that surely resonates with a wider audience; we now use it in the Introduction, alongside with a relevant reference that introduces the scalar property as "Weber's law" for interval timing.

In general, as was mentioned by Reviewer #3, I think the manuscript still is written in a form that often mixes broad assumptions about the reader's knowledge with overly complex and technical language—but unnecessarily so for the purpose.

While a complete solution would likely involve restructuring the manuscript to separate the core ideas and results from the technical material, it would suffice to at least have a set of colleagues who are not familiar with the specifics to note all the places where clarifications or simplifications would benefit the reader. I don't feel that it is the reviewers jobs to do copy editing, so I will not provide that level of detail, but I will point out a few specific problems I think should be addressed.

First, 'Shadlen-like' as a description of the class of experiments is both not technically correct nor clear to a reader unfamiliar with the specifics of those experiments, while the model and general points are widely applicable. The correct citation for the specific random dot kinematogram would be Newsome & Pare (1988) or perhaps Newsome, Britten & Movshon (1989) along with the related papers. However, the general question of integrating sensory information is much larger and most of the results in the paper extend beyond the narrow MT/LIP random dot kinematogram task literature. The same task has been used extensively and similar tasks with different superficial structure show the same basic behavioural signatures e.g. in olfaction, somatosensation, etc. In short, it would be better to describe the class of tasks and mention the specific experiment you replicate rather than using a reference that makes assumptions of the reader's knowledge and implies perhaps a narrower range of applicability.

We replaced “Shadlen-like” with more descriptive expressions (and we now cite *Newsome & Pare (1988)* when introducing the random dot experimental setup). We now rephrase some passages in order to convey the idea that our agent is intended as a model for a general class of problems in perceptual decision making (and added a new reference, *Heekeren et al. (2008)*, to even more emphasise the point).

It would be nice if Figure 1 graphically described the complete model. By this I mean that the choice (multiple-logistic/Boltzman/softmax) function is missing. As is the a graphical description of the environment’s relation to choice and reward feedback (i.e. consider the corresponding actor-critic agent graphic from Sutton and Barto). While the current figure is sufficient, a reader wanting to understand the model clearly needs more.

We have changed Figure 1 to include additional details about the task and the model.

Figure 2 seems like it might be clearer to the reader in the context of the rest of the paper if the non-exponentiated (i.e. summed weights, rather than probabilities) were used. This would also correspond more to the scale used for the colored circles.

In this case, we prefer showing the probabilities to emphasise the probabilistic nature of the proposed model. Examples of weighted signal integration for a single episode can be found in Fig. 9.

Signal neutrality is an unclear term that is not found in the literature nor is it easy to interpret in the context as a natural phrase in English. The signal is the external property (i.e. the random dots) and not the variable represented by the accumulators or neurons. I believe the authors intend to imply ‘signal strength neutrality’, that is, that the coherence differences are no longer observed in the internal variable at the time of decision—regardless of whether it is approaching an absorbing boundary or if it is caused by the mixing of time constants in their model. I don’t want to propose a specific solution for the authors, but perhaps they could find a term from the literature or at least introduce this term very clearly at the conceptual level. Currently, it’s introduced in a narrow context of neural signals. The same properties are seen in other decision making models (e.g. Wang 2002). Specifically, regardless of the terminology, clarification of the relationship between the way the author’s model implements a decision and the others and the respective models’ relationship to this property should be more clear to the reader. For example, the neurons are thought to be reaching a saturation (perhaps maximal firing rate) prior to motor command initiation which resets in some interpretations. Other models predict this property trivially (in that they define the decision as reaching a common threshold. The criteria and its relationship to models and the data should be clear along with the implications.

As we now state in the Introduction, “signal neutrality” is a shorthand we use to indicate a specific (but well replicated) experimental finding. We made an honest effort to find a term in the literature for that, but we found nothing. Then we devised a very brief (and, therefore, very imprecise and, admittedly, not self-explicative) description of the phenomenon - “signal neutrality”; meaning that there is some variable that carries information about the decision and that, in proximity of the decision, does not distinguish between (it is neutral with respect

to) different levels of the signal-to-noise ratio of the external stimulus. As signal in our case has, in many respects, the same meaning of signal in “signal-to-noise”, we think that using “signal strength” would make the text heavier without adding too much in clarity.

We now rephrased for better clarity, in the Introduction, the description of the experimental findings we refer to with “signal neutrality”, adding two relevant references.

We are not aware of other modelling works tackling the issue directly; in particular, Wang 2002, as far as we can understand, deals with a task in which decision is delayed after a fixed duration of the stimulus; what the author finds is that after the stimulus is turned off, the population of neurons reaches a stationary firing rate that is independent of the signal-strength (due to attractor-like dynamics); but there is no ramping activity anymore (as observed experimentally and in the proposed model).

In this respect, we want to underline (and we tried to make it clearer in the text at several points) that signal neutrality refers to a collapse of some key decision variable for several hundred of milliseconds before the decision, not just at decision time - something that, of course, would be automatically granted by any model with a deterministic decision threshold.

It is unclear why the signal neutrality property drops at the end of training and how flat the changes in expected reward are at that point. Is this a possible overfitting or other problem with the reward gradient? Is the model actually still improving while signal neutrality is declining for a reason that can be explained? The current text does not provide much insight and seems to imply that the model has not asymptoted (“very modest performance gains”), but it’s unclear then how to interpret if the signal neutrality property is being appropriately related to the monkeys (asymptotic) performance, and more critically, just what is changing that allows the performance gains at the expense of the signal neutrality. I think there is no particular justification provided for the length of training (or point selected along the relative flat performance period) for analysis. Nor does it seem necessarily pertinent to the story, so perhaps Figure 7 and the accompanying text could be pushed to supplementary information or at least moved to section 3.6 with Figure 10.

We now stress in the text that with “modest performance gains” we mean an increase in performance of about 1% after an additional *900.000* training episodes. In this later phase of training, signal neutrality starts to erode. We don’t have a simple explanation for that; the message that we now try to better convey in the manuscript is: trying to be “signal neutral” is a good strategy (we call it a “satisficing” one) to solve the decision problem - good but not optimal - when you have a very volatile environment (like in our case, with a two-level uncertainty: the signal noise and the variance of the noise itself).

Related to this line of reasoning and in line with the reviewer's remark, we now advance an hypothesis on why animal subjects display signal neutrality.

We thank the reviewer for the suggestion that the decline of signal neutrality at later stages of training could signal some kind of overfitting, in the sense that when signal neutrality breaks probably the agent, confronted with changes in the statistics of the environment (like

a change in p(mu)), will require many more new training episodes to adapt. This is a very interesting hypothesis we plan to test in the future (we have inserted a comment on that in the manuscript).

Mention Weber law and Weber scaling when introducing and contextualizing the scalar property. I note the word "glaringly" in the abstract when describing this which seems quite odd. But more generally, this is a broad and important literature of observed properties (with varying underlying models) that should and could be at least contextualized with the 'scalar property' used here.

We removed "glaringly" in the Abstract, and now we mention Weber's law.

The description of collapsing boundary property and Figure 8 is a bit confusing for the reader as presented.

In Figure 8, the propensity from the clock follows the expected (near optimal) shape of the collapsing bound, but the actual decisions made by the agent are presented showing the signal evidence, which is closer to a flat distribution of evidence—if I'm following the subscripts correctly and the filled circles only represent the stimulus weights for the choice. But this is what you would expect from the combination, and the description of the clocks providing a 'soft' bound effect.

However, the text seems to imply that the actual sensory evidence weights are also showing the effect, which seems counterintuitive — as they have been mathematically separated in the variable shown to exclude the effects of time, other than via the distribution of integrator time constants.

We tried to clarify this point in the paper. The decision-making process is a combination of signal integration and the perception of time defined through the clock. As the reviewer pointed out, the clock acts as a soft threshold mechanism that is independent from the external signal s(t) by construction. The average of the clock across time shapes the collapsing boundaries, depicted through the black line in Figure 8. To confirm that this observable acts as a soft threshold mechanism, we showed that decisions occur when the weighted signal integration is close to the boundary inferred by the clock. The grey area is the region where the majority of decisions are made and follows the trend of the collapsing boundaries, thus demonstrating that the latter can be treated as a soft threshold.

Sensory evidence is not sufficient to reproduce the 'desirable' shape of the soft threshold. Indeed, we show in panel C that the model without a clock mechanism is unable to learn the collapsing boundaries.

In the end, they vary slightly across time in the direction you might expect from the optimal POMDP model, or the clocks alone. But if I were to take the mean decision points (inferred from the 80% shaded band), the weighted evidence would barefly change with time compared to the variance of the decision times. It's unclear from the description and the few examples shown (and the 80% band) if the pure sensory evidence weighted integrators are an additional contribution to the soft collapsing bound property or if it would be better explained (or at least described) as a model that has a collapsing bound propensity via an effectively separate mechanism from the sensory evidence?

The model actually has to separate mechanisms for integrating sensory evidence and the passage of time. The time integrators, we argue, implement a moving threshold (collapsing for long times) mechanism that the sensory evidence (as expressed by the weighted sum of sensory integrators) has to pass in order to make a decision. As our model is probabilistic in nature, there is no real deterministic threshold; yet one can define a “soft-threshold” as the region where you can find the sensory evidence when a decision is made - this is basically the 80% shaded band. We agree with the reviewer that the range of variation of the moving threshold is not large as compared to the average width of the 80%-band; yet, the variation is systematic. Furthermore, even if we don’t show any structured comparisons, there is a clear impact of the time integrators on the performance of the agent.

In which case, it’s not clear what the samples and 80% band is conveying. I also note that the impact of the time on the decision times given the parameters used in the current simulations may be small. I assume that different parameters (e.g. reward function) or modeling a different task variant (e.g. one with an opt-out as seen in the literature) might demonstrate the effect of this propensity more clearly. However, this seems likely just the way the bound is described relative to the model’s decision points in the figure. The comparisons are interesting and the main point seems to hold as long as the clock integrators show the curves presented in the black lines.

Please, see the reply above.

In Figure 9D, isn’t there a natural prediction (e.g. from the optimal POMDP or relative to a fixed sumed clock from 9C) that would provide true predictions rather than the polynomial fits? Or, to put it another way, shouldn’t the function of the weights be smooth over the times for for large samples so that you see a clear peak that shifts? This may be just a function of the noise relative to the number of models trained or the amount of data from the model?

We believe that there has been a misunderstanding, the x-axis does not correspond to the time in the episode, but to the timescales of the integrators. The panel is showing the optimised value of the parameters \theta (signal integration) as the timescale changes, reflecting the importance that the agent gives to the different integration times. The results are obtained after optimization, and true predictions are not available. Otherwise, we would have known the solution to the task a priori.

The effect shown is not due to noise, and the shift of the relative peak can be also understood by analysing the performance of the models that exploit a single integrator (Panel B). Indeed, in panel B we have shown how the most ‘effective’ timescale becomes lower as the intrinsic noise increases (see how the performance of the model with \tau=10s drops in comparison to the faster one from left to right of panel B). Similarly, the model is learning to give more emphasis to faster timescales (thus the shift) as the intrinsic noise raises.

Figure 10 needs a matching color scheme or legend for D,E,F.

We have used a scale of greys as a matching colour scheme. To emphasise this, we have also adopted different line styles.

Overall, the learning results are interesting, however they suggest to me (i.e. F7 and F10) that the model struggles a bit to converge and is perhaps taking a trajectory that is caused

by the lack of constraints on the weights. Reviewer #1 also asked about the weights and you responses suggests you didn't consider the fact that the weights necessarily are only relative—that is, while they can vary across the whole number line in your current definition, by the constraints of the learning algorithm the critic weights are implicitly constrained to rescale the average evidence and clock integrators to the scale of the reward. On the policy side, there is no such implicit constraint as the relative weights enter into the softmax in a form where they can trade off across the (absolute) scale. Given that for the data the agent sees and the noise in the integrators and signal, it might be better for the study of learning to constrain the actor (policy) weights to sum to one (or zero) and always re-normalize them to a unit vector. This would ensure the reward gradient is driving the relative weighting. As the reward function is trivial and the basis set large, perhaps a similar consideration for the critic is appropriate. In any case, I would probably move the learning to supplementary if left as is, despite it being interesting and generally supportive of the overall story.

The reviewer is right in pointing out that the set of parameters we chose is redundant; think for example of the three biases b_left, b_right, and b_wait: only two combinations of them (e.g., b_right - b_wait, b_left - b_wait) have a real effect on the model, because of how they enter the definition of the choice probabilities (Eq. 17 in the manuscript). We now underline this important fact when describing the model.

Yet, normalising the weights - for example by fixing the sum of their absolute values to a constant, though useful for illustrative purposes (as in Fig. 9D and E, for example), would severely impair the agent, imposing a quite unnatural constraint on the range of its possible behaviours. Note for example that multiplying all the parameters in the model for a factor tending to infinite would make the probabilities p_right, p_left, and p_wait either 0 or 1 at every time, thus rendering the agent practically deterministic. This possibility is clearly cut out by the normalisation of weights.

We moved the Learning section to the appendix.

Check section 5.2, F.igure 12 is referenced when Figure 13 is meant throughout the text. The (actual) Figure 12 would be more useful if it showed 1-21 integrators, as above 21 there isn't any information and given the task, seeing the degradation to 1 integrator is more useful. Similarly max time scale from sub 1 to 10 would be more informative.

We fixed the reference, we don't show more than 21 integrators in the figure in the main text. Only in appendix, for completeness.

Finally, overall the technical notation and choice of language in describing the model mixed with connections to the literature and experimental data makes the readers job difficult. When you read much of the manuscript and look at the figures, you need to refer to the specific definitions of the objects and the authors choice of symbols, etc. It was already noted in the first reviews that the language and copy editing should be checked and that it was difficult. This seems unnecessary, in that in many places the technical elements—that is the mathematical formalism and choice of notation—are not that critical to any of the author's points.

We have proofread the manuscript (also following the reviewers' remarks). Even though we disagree on the fact that technical elements and precise definitions of what is shown are not critical for understanding the results, we are open to any other suggestions to improve the technical notation and make the various definitions more intuitive for the reader.

Further, as there is no analytical elements to the paper, and the core models and definitions are common enough in the computational neuroscience literature and the surrounding referenced literature, from this reviewers point of view, much of the technical definitional math could be separated from the descriptions of the computation and algorithms and the conceptual objects and where possible. The use of words rather than symbols for non-canonical elements—at least in the main text—for example. As an example, there are 10 integrators and 10 clocks. Why "x_{tau}(t)" and "x+{tau}^{Tau}(t)"? Why not "integrator_{tau}(t)" and "clock_{tau}(t)"?

Although we are conscious that our model is quite simple when compared to others in the computational neuroscience literature, we strongly believe that a clear mathematical definition of all the variables is paramount for a real understanding of less technical concepts (the more so in a Journal like PLOS Computational Biology). For this reason, we decided to keep the technical definitions in the Methods section.

Following the reviewer's remarks, we changed the superscript for symbols related to time integration from T to C (remarking that C stands for "clock") and we introduced the superscript s (for ''signal'') for all the components related to signal integration. Moreover, we changed the notation for the parameters, naming \theta the parameters for the actor and W the parameters of the critic as in standard reinforcement learning textbooks.

If the authors want to make their ideas accessible to a wide audience, they can make it easier for them without diminishing the technical clarity for those who wish to evaluate the details. After all, supplementary information could be used and the code is shared—in fact, Jupyter notebooks include latex rendering so the full math should be there too. However, this is as much a personal style comment and a suggestion for trying to increase the audience and impact as a criticism. The current draft is an acceptable presentation of the ideas and results.

Which brings me to the final comment, the Jypter notebook now in the code repository is a vast improvement, but the primary code and the notebook could both provide exactly the level of beneficial clarity requested above by including actual comments and clearly explicating text cells. Why the authors seem intent on providing all the code but making exploring the model difficult is a mystery.

We disagree with the reviewer: we definitely don't want to make exploring the model difficult. We believe in open science and we gladly provide the whole code, both for simulation and data analysis. We made what we think is an honest effort to make the code usable, by - in the first place - writing a readable code to the best of our skills, by making parameters'

names as compatible as we could with the symbols in the paper, by providing working examples that reproduce key results presented in the paper. And we will be more than pleased to offer assistance to anyone interested in using the code.

Anyway, we have now expanded the comments in the companion Jupyter notebook, to make it more readily accessible to the interested reader.

We thank the reviewer for the suggestions and the analysis of our manuscript.

Reviewer #3

Thank you for the revision. Although the manuscript would still benefit from proof-reading (example, line 99), I think the manuscript has been improved much in terms of clarity, together with other reviewer's comments. My comments have been fully accounted for.

We proof-read the manuscript, also in the light of the reviewers’ suggestion. We thank the reviewer for his/her/their kind remarks.

We thank the reviewer.
