# Supplementary material for: Signal neutrality, scalar property, and collapsing boundaries as consequences of a learned multi-timescale strategy
Source: PLoS Comput Biol. 2022 Aug 5;18(8):e1009393. doi: 10.1371/journal.pcbi.1009393 (PMC9462745; doi:10.1371/journal.pcbi.1009393)
Supplement: Attachment — Submitted filename: review.docx [file pcbi.1009393.s005.docx]

**Prologue**

I thank the editor and authors for the opportunity to review this interesting paper. Although I enjoyed reading about the idea, the process was made difficult with the figures all at the back of a *very, very* long manuscript. For future revisions please insert the figures where they belong along with the legends within the main text.

**Summary**

The authors developed a perceptual decision-making model which consists of multiple time-scale accumulators as compared to the typical single time-scale accumulator model such as the drift-diffusion model. The authors trained this model through reinforcement learning, so that it can come up with an effective decision-making strategy which can deal with highly volatile environments. Using simulation, they find that the model produces typical features similar to experimental findings from single-neuron decision-making studies such as signal neutrality, scalar properties and collapsing bounds. The authors attribute the manifestation of these properties to the multiple time-scale accumulator structure, suggesting it to be a critical feature for decision-making.

The topic is relevant and interesting, and the idea of a multiple, parallel time-scale accumulator structure in the brain working together to deal with the temporally dynamic environment sounds intuitively plausible and intriguing. The concept of multiple time-scales in human perception is also important and has gained much attention, therefore expanding a typical accumulator model to incorporate multiple time-scales seems timely. The model seems original and interesting as it attempts to focus on key features observed in real data. However, I find the paper not so easy to comprehend and thus it can be improved in terms of writing and presentation, as well as elaborating more on why their model and its replication of some features in experimental data is so critical in understanding perceptual decision-making. Additionally, some of the results seem to warrant more explanation. Crucially, without testing the model on real data, I find the paper to be somewhat incomplete, albeit with potential. Or at least if the simulations were done for different kinds of simulated tasks dealing with stimuli with actual multiple time-scales, perhaps it can highlight more the strength of the model. Below are specific comments and questions about the paper.

**Introduction-related comments**

In page 5, the authors write that signal neutrality, scalar property, and collapsing bounds are the hallmarks of an optimal strategy in decision-making. However, they do not explain why this is. It seems the authors are basing the validity of their model on showing that these three properties can be reproduced from the proposed model, specifically due to the multi-time-scale accumulators. Therefore, it seems critical to first convince the readers that these properties are indeed crucial to optimal decision-making in the introduction (i.e., L118-L121: sentence difficult to understand).

**Results/Methods-related comments**

- 2.1 Model description: It seems the random dots’ coherence is modeled as the mean value of a signal with mu. It says the value of mu is resampled for each episode. Was the distribution continuous? How many values could mu assume throughout the training? If the mean was zero in a gaussian, then most of the trials were quite difficult? How did this affect the training?
- If the model’s strength is to have multiple time-scale accumulators, why wasn’t the input stimuli composed of multiple time-scales as well? For example, using different sampling rates of the signal or using signal of which coherence is changing within a trial, etc. may be able to highlight the model’s strength more.
- The symbols and variables are not explained at first appearance; xi should be explained at L152, not L160. What is the role (meaning) of b?
- L165: In the figure, the white circles alone do not seem to be enough to explain the softmax combination. A more descriptive cartoon may help. Same for V(t) in L185-L187. The figure doesn’t add much information about what the text is meaning.
- 2.2 Decision as a majority vote: this part seems to be key to highlight the novelty of the model. Yet, how the leaky integrators with such different time-scales align at the same time point to create bursts of probabilities is not clear. How does a 10 s accumulator already have such high probability after only 400 ms after trial onset? (point 2 in Fig 2). Also, from Fig 2 it seems decisions are made at points 1 and 2. Does this mean there were two decisions within an episode of 1.6 s?
- Figure 3C: What are the white dots? In the text it says that the parameters of the curve were fitted to experimental data. Does this mean the authors obtained experimental data from reference 4? It is not clear what actually has been done to produce Figure 3C. Please clarify.
- Figure 4D: Figure shows that agent shows the exact same signal neutrality and accuracy for all taus. What does this mean? Please explain.
- In section 2.5, the authors show that the scalar property and signal neutrality both share the same origin, namely the multiple time-scales present in the model. It would help the reader not so familiar with the technical derivation if there was a simpler explanation why multiple time-scales leads to both these features (L387-L390).
- Figure 10A: What is the reason for the four time-courses suddenly diverging before 10^2?

**Writing-related comments**

I think the writing can be overall improved to be clearer and simpler, by using plainer sentences and easier words, considering many if not the majority of readers are probably non-native speakers.

Here are some examples with suggestions in red font:

Second line of the abstract: “We endow..”, this sentence can be simpler, e.g., “We introduce/developed a probabilistic agent comprising .. with a wide range of time-scales.. to weight differently filtered versions of a noisy signal”. Then the next sentence, “.. according to which a decision is made when the accumulated evidence hits..”. Also the next sentence “The agent instead decides during short, transient windows, when these multiple integrators line up, similar to a majority vote”. Maybe this sentence and the previous sentence can be combined, to make it clear what the author’s new model does differently than the standard accumulator model. Also, the next point 1): here, the authors write ‘signal coherence’ without first saying anything about the task, therefore what coherence means here is vague. Point 2): perhaps an easier word such as clearly instead of ‘glaringly’ would be better. Again, authors talk about signal coherence without introducing it. Simply including a short sentence about what task has been used before introducing what the new model can do would help. Second to last sentence of abstract: “.., which emerge from the interaction of a multi-time-scale learning agent with a highly volatile environment, are hallmarks of an optimal decision strategy ..”. There are many examples like this throughout the text, and I recommend trying to make the sentences plainer and clearer for the reader, since the content itself is already quite technical.

Also, some sentences (e.g., L556-L558) seem to be incomplete, without a subject. These need to be re-written in order to accurately convey what the authors would like to say. In addition, often the authors cite a paper without sufficiently describing the citing content (e.g., L584). It would help the reader if the text is written more independently of the references.

**Editing-related comments**

- Please choose either timescales or time scales (or time-scales).
- I think putting an ‘s’ after the x(t) to indicate plural is strange, since x(t) is not a word. Perhaps simply x(t) may suffice.
- Figure 1: it seems the two x(t) in the figure are flipped).
- L430: then -> than. More cases like this throughout text.
- Figure 3: ‘agent’ and ‘model’ seems to be used interchangeably. If that is the case, please choose one, since it is confusing.
- Legends missing for the colors in Figure 8.
